# Round the Hospitals

**Published:** 1916-09-30

**Authors:** 


### ROUND THE HOSPITALS.

LAST week, when discussing the Committee appointed by the War Office to advise on the Supply of Nurses, several criticisms were offered upon the action of the authorities. Those observations were purposely expressed in temperate language in order to make a graceful retreat from an impossible position as easy as might be for the War Minister, who had been obviously ill-advised by someone in his department. Even before THE HOSPITAL was distributed last week, though some hours after it went to press, the chairman of the Committee (Mr. W. Bridgeman, M.P.) represented to Mr. Lloyd George that the Committee as constituted could not be of any effect. The fact was that the indignation of the nursing profession, and not of that profession alone, had already become so vehement that the Committee's position was untenable. Other papers, especially the *Daily Telegraph* among the lay press, had used language much more forcible than that of THE HOSPITAL; although there was every excuse for sweeping criticism, it is perhaps regrettable for the reason

* Previous articles appeared on August 26 and September 16.

already mentioned. At any rate the errors of commission in the *personnel* of the Committee have not been rectified, even if those of omission are in a fair way to be so. Meanwhile the College of Nursing has put a spoke in the wheel by addressing a letter to Mr. Lloyd George "expressing a hope that he would suspend action in connection with the Committee until the matter has received further consideration." This prompt action by the College should go far—particularly if its advice is accepted—to convince nurses all over the kingdom that the College is watchful of their interests, and able and willing to protect them.

Matrons of hospitals all over the country are doing useful service for the College of Nursing and for the nursing profession in summoning meetings to discuss the College and the Registration Bill. Several such meetings have been held lately, which have been well attended by representative members of the nursing profession who have displayed a keen interest in the questions discussed. At Plymouth last week, where the meeting was convened by Miss Tait McKay, matron of the 4th Southern General Hospital, Miss Rundle pointed out that it depended on the nurses how soon the Registration Bill would be brought before Parliament. The Council of the College have met the Central Committee for the State Registration of Nurses with the view of arriving at an agreed Bill, and it was advisable that they should go to Parliament with a very large register of nurses who have become members of the College as a guarantee of their desire for instant legislation. It would be the duty of the College of Nursing to see that legislation procures for the profession that standard which its members desire. With reference to the question of the V.A.D. members, Miss Rundle laid stress on the point that there should be some controlling power, and that that control should be vested in an institution of trained nurses. During the past two years V.A.D. members had done good and successful work when placed under the control of the profession. Miss Gibson pointed to the educational value of the College, which, under the Registration Bill, would settle a definite curriculum of training for all nurses who might desire to come on the register at the end of the period of three years' grace, during which any person who is qualified for registration under the conditions laid down by the College of Nursing (see The Hospital, June 10, p. 237) may be entered on the register without being required to pass an examination. It was therefore to the advantage of all trained nurses to join at once.

We hope our readers have studied the articles on "Human Temperaments," by one of the ablest and most penetrating writers of the day, which we have been publishing during the last few months. That on "The Envious Temperament," which found its place as No. IX. in the series (see The Hospital of August 19, p. 471), should have a special interest to those of us who may find it difficult to realise why it is that the best work and the best workers are sometimes persistently spoken against by some who are ambitious of authority in the hospital world. We have recorded the excellent work done at St. Thomas's Hospital by the organisation and successful administration not only in the wards set apart for the wounded soldiers, but in the special accommodation provided in military huts in the grounds. The staffing arrangements, comfort, amusements, and general organisation and work in connection with the accommodation for military cases and its results at St. Thomas's Hospital are amongst the best products of the war's demands. Why is it, then, that there are people to be met with, who ought to know better, who shake their heads and assume an attitude of disapproval when the work of St. Thomas's Hospital is mentioned? These things ought not to be in the hospital world, where the very nature of the work should produce higher ideals and standards of conduct, with a just appreciation of other men's work. It is remarkable that any persons dominated by the envious temperament should manage to live and to remain persistently in the hospital field.

A complaint reaches us that certain leading medical women who in the past have done much excellent work seem to be utterly blind to the value of trained nursing. In one hospital the nursing sisters, many of them experienced and highly trained women, are said to be treated with the scantest courtesy. It is reported that highly-placed medical women repeatedly tell the girl-orderlies that they know as much as the sisters, that girls with a few weeks' experience are left in charge of wards for as long as four weeks at a time, and that it is openly said that the untrained helpers are more suitable for such responsibility than trained sisters. We hope the College of Nursing will black-list this hospital and others, where the ward sisters receive so little support from those in authority as to render their position an impossible one. We understand that in one of the great Nursing Services no V.A.D.s are accepted who have received training at such a hospital, as it is well known that the girl-orderlies have been taught to be insubordinate to the nursing sisters.

Miss Bennett, the matron, may well be proud of the result of the sale of work held last week in the playground of the military section of the Metropolitan Hospital in Kingsland Road, the object of which was to reduce the debt on the hospital and to provide extra comforts for the wounded soldiers. Every nurse and many of the patients contributed to the stalls, whilst the local residents proved their appreciation of the work carried on in their neighbourhood by presenting the materials for tea, providing articles for the stalls, or collecting money on behalf of the objects of the sale of work. In fact the co-operation was so general and spontaneous as almost to resolve itself into a friendly competition between the helpers for the first place, with the result that no less a sum than £360 was realised by the day's sale, under £6 of which went in expenses. Truly a splendid and encouraging result.

For Matrons' and Sisters Appointments, see p. v.

